# Bioprocessing of “Hair Waste” by *Paecilomyces lilacinus* as a Source of a Bleach-Stable, Alkaline, and Thermostable Keratinase with Potential Application as a Laundry Detergent Additive: Characterization and Wash Performance Analysis

**DOI:** 10.1155/2012/369308

**Published:** 2012-12-17

**Authors:** Ivana A. Cavello, Roque A. Hours, Sebastián F. Cavalitto

**Affiliations:** Research and Development Center for Industrial Fermentations (CINDEFI) (UNLP, CONICET La Plata), Calle 47 y 115, B1900ASH La Plata, Argentina

## Abstract

*Paecilomyces lilacinus* (Thom) Samson LPS 876, a locally isolated fungal strain, was grown on minimal mineral medium containing “hair waste,” a residue from the hair-saving unhairing process, and produced a protease with keratinolytic activity. This enzyme was biochemically characterized. The optimum reaction conditions, determined with a response surface methodology, were 60°C and pH 6.0. It was remarkably stable in a wide range of pHs and temperatures. Addition of Ca^2+^, Mg^2+^, or sorbitol was found to be effective in increasing thermal stability of the protease. PMSF and Hg^2+^ inhibited the proteolytic activity indicating the presence of a thiol-dependent serine protease. It showed high stability toward surfactants, bleaching agents, and solvents. It was also compatible with commercial detergents (7 mg/mL) such as Ariel, Skip, Drive, and Ace, retaining more than 70% of its proteolytic activity in all detergents after 1 h of incubation at 40°C. Wash performance analysis revealed that this protease could effectively remove blood stains. From these properties, this enzyme may be considered as a potential candidate for future use in biotechnological processes, as well as in the formulation of laundry detergents.

## 1. Introduction

Microbial proteases are the most widely exploited industrial enzymes with major application in detergent formulations [1, 2]. These enzymes are being widely used in detergent industry since their introduction in 1914 as detergent additive. Over the past 30 years, the importance of proteases in detergents has changed from being the minor additives to being the key ingredients. The main areas where use of proteases has expanded are household laundry, automatic dishwashers, and industrial and institutional cleaning. In laundry detergents, protein stains such as grass, blood, food, and human swear, are removed through proteolysis. The performance of proteases is influenced by several factors such as pH of detergent, ionic strength, wash temperature, detergent composition, bleach systems, and mechanical handling. Thus, the key challenge for the use of enzymes in detergents is their stability. Various attempts have been made to enhance stability of alkaline proteases by site-directed mutagenesis [3] and protein engineering. “Subtilisin Carlsberg” has been protein engineered to obtain a bleach-stable, alkaline protease by molecular modification [4], but still, there is always a need for newer thermostable alkaline proteases which can withstand bleaching agents present in detergent. Among these different proteases, keratinases constitute a group of enzymes capable of disrupting the highly stable keratin structure consisting of disulfide, hydrogen, and hydrophobic bonds in the form of *α*-helices and *β*-sheets [5].

Argentine's economy has traditionally been based on agriculture and related industries. Livestock (cattle, sheep, and poultry) and grains have long been the bulwark of its wealth; its cattle herds are among the world's finest. There are more than 50 million of livestock which generate large amounts of waste including insoluble keratin-containing animal material such as feather, hair, wool, nails claws, hooves, horns, and beaks.

Although hair-saving unhairing processes reduce the organic load from beamhouse liquid effluent, a new solid residue called “hair waste” is generated, its appropriate disposal being then necessary. In a hair saving unhairing process almost 10% (wet basis) in weight of each salted bovine hide become hair waste. Because of tanning industry in Argentina processes, on average, more than 100 ton of salted hide per day, more than 10 ton of solid waste are generated per day, generating an environmental problem of considerable magnitud [6]. Since it is a protein waste, it deserves special attention in order to be utilized for practical purposes.

The fungal biotransformation of the “hair waste” implies considering it as a raw material instead of the present idea of disposability. Thus, hair waste would be the substrate, on which the fungus would act, giving rise to a (partially) hydrolyzed protein with different potential uses (i.e., as animal feed, fertilizer, etc.). In addition, the fungus would produce a proteolytic (keratinolytic) extract of biotechnological interest with a variety of potential applications (cosmetics, textiles, detergent industries, etc.). This paper deals with a particularly case of the second aspect above mentioned.

A series of studies dealing with the bioconversion of keratin waste resulted in the discovery of a novel keratinase activity in a culture supernatant of a fungal strain (*Paecilomyces lilacinus* (Thom) Samson LPS 876) grown on chicken feather as a sole of carbon, nitrogen, and energy source [7]. In this paper, we report the biochemical characterization, including the effect of some surfactants and bleaching agents on enzyme stability, its compatibility with various commercial liquid and solid detergents and a study of an efficient stabilization method toward heat inactivation, of the keratinase produced by *Paecilomyces lilacinus* growing on hair waste substrate. 

A wash performance was also done with particular emphasis on its potential application as an enzyme ingredient for the formulation of laundry detergents.

## 2. Material and Methods

### 2.1. Microorganism and Identification as a Keratinolytic Fungus

*Paecilomyces lilacinus* (Thom) Samson LPS 876, a nonpathogenic fungal strain locally isolated from alkaline forest soils, was used. It was selected from Spegazzini Institute Fungal Culture Collection (La Plata National University, Argentina) after a preliminary screening for keratinolytic fungal strains on feather meal agar containing (per liter) the following: defatted chicken feather meal, 15 g; NaCl, 0.5 g; K_2_HPO_4_, 0.3 g; KH_2_PO_4_, 0.4 g; agar, 15 g, pH 7.2. The strain selected was punctual streaked and incubated at 28°C for 15 days. The growth of the colony and the clear zone formation around it were daily studied. The ability to degrade keratin was determined according to the presence or absence of hydrolysis halo [8].

### 2.2. Culture Conditions and Enzyme Production

Production of protease by* P. lilacinus* was carried out in a minimal mineral medium containing (per liter) the following: 10 g hair waste, 0.496 g NaH_2_PO_4_, 2.486 g K_2_HPO_4_, 0.016 g FeCl_3_·6 H_2_O, 0.013 g ZnCl_2_, 0.010 g MgCl_2_, and 0.11 mg CaCl_2_. Hair waste, obtained from a local tannery, was washed extensively with tap water, dried at 60°C for 2 days, and then kept at room temperature until used. In all cultures, it was a sole carbon, nitrogen, and energy source. The pH was adjusted to 7.0 previous to sterilization [9]. Cultures were performed at 28°C and 200 rpm for 10 days in an orbital shaker, in 500 mL Erlenmeyer flasks containing 200 mL of medium, inoculated with 2 × 10^6^ conidia per mL. Samples of 5 mL were withdrawn at regular intervals, centrifuged (3,000 ×g, 10 min, 4°C), and the supernatant was used for pH, protein content, and enzyme activities determinations.

### 2.3. Protein Determination

The protein content was determined by Bradford's method using bovine albumin fraction V (SIGMA) as a standard [10].

### 2.4. Protease Activity

Proteolytic activity was measured as described by Liggieri et al. [11] with some modifications. Reaction mixture containing 100 *μ*L of appropriately diluted enzyme preparation and 250 *μ*L of 1% (w/v) azocasein solution in 0.1 M Tris-HCl buffer (pH: 9) was incubated for 30 min at 37°C. Reaction was stopped by precipitation of the residual substrate with 1 mL of trichloroacetic acid (TCA, 10%). The mixture was kept at room temperature for 15 min and then centrifuged at 3,000 ×g (10 min, 20°C). One mL of 1 M NaOH was added to 1 mL of the supernatant, and absorbance was measured at 440 nm. Measurement was made in triplicate using a blank with a heat inactivated enzyme solution. One unit of proteolytic activity (U_C_) was defined as the amount of enzyme that, under test conditions, causes an increase of 0.1 units in the absorbance at 440 nm per minute. Azocasein was synthesized as described by Riffel et al. [12].

### 2.5. Keratinase Activity

Keratinolytic activity was assayed as described by Joshi et al. [13] with some modifications: 800 *μ*L 0.1 M Tris-HCl buffer (pH: 9) was added to 30 mg of azokeratin, and mixture was stirred for 15 min at room temperature until the azokeratin was completely suspended. Appropriately diluted enzyme preparation (100 *μ*L) was added and incubated for 25 min at 37°C with individual magnetic stirring. Reaction was then stopped by the addition of 200 *μ*L of TCA (10%) and centrifuged at 3,000 ×g (10 min, 20°C). The absorbance of the supernatant was measured at 440 nm. A blank was prepared using heat-inactivated enzyme preparation. One unit of keratinase activity (U_K_) was defined as the amount of enzyme that, under test conditions, causes an increase of 0.01 units in the absorbance at 440 nm per minute. Azokeratin was synthesized as described by Joshi et al. [13] using defatted feather meal as keratin source.

The relationship between keratinolytic and proteinolytic activity (called K : C ratio) is widely used as a parameter for evaluation of the keratinolytic potential of proteases [14, 15]. K : C ratio of *P. lilacinus*' keratinase was compared with those of several commercial proteases such as proteinase-K (Promega), Alcamax (Cergen), and papain (FLUKA). Stock solutions of these commercial enzymes, in concentration of 1 mg/mL, were prepared in distilled water. They were diluted adequately, and both enzyme activities (keratinase and protease) were determined as described above.

### 2.6. Biochemical Characterization

A supernatant of a 5-day-old culture was used as crude enzyme preparation for the biochemical characterization of the keratinases produced by *P. lilacinus *(2.5 U_C_/mL). It is worth to mention that, for practical reasons, protease activity was used in our research to represent keratinase activity, since keratinolytic (azokeratin) and proteolytic (azocasein) activities are directly related [16], a fact that was also later confirmed for *P. lilacinus* keratinase (see below).

### 2.7. Effect of pH and Temperature on Enzyme Stability and Activity

The effect of pH and temperature on enzyme activity and stability was studied using a Response Surface Methodology (RSM) based on the use of a matrix of experiments by which the simultaneous variations of the factors can be studied. Uniform shell design proposed by Doehlert was selected for design the response surface [17]. The main advantage of this procedure lies in the possibility of extending this uniform net in any direction and increasing the number of factors in the study. The real values of the independent variables were coded based on a linear functionality between codified (*Z*) and actual values (*X*) according to:

(1) X:Z∗ΔXΔZ+X0,

where *X*_0_ is the real value of the central point and ΔX and Δ*Z* are the difference between the highest and lowest values of real and coded numbers, respectively.

Multiple regression analysis based on the least square method was performed using Mathcad 2001 software [17]. For both determinations, the central values (zero level) for the experimental designs were pH 7.5 and temperature 40°C.

The pH and thermal stability were evaluated incubating the enzyme preparation for 2 h at each chosen experimental condition. The residual protease activity was determined under standard conditions and expressed as percentage of residual activity relative to a control (measured at 0 h of incubation). Temperature varied between 20 and 60°C and pH between 3.0 and 12.0, using a mixture of buffers (Glicine, MES and Tris, 20 mM each).

The protective effect of CaCl_2_ and MgCl_2_ (5 mM, each) and sorbitol (10% w/v) on heat inactivation was also studied. The crude enzyme was incubated at 50–60°C with and without the chemicals mentioned above, and residual enzyme activity was measured at regular intervals under standard assay conditions.

The effect of pH and temperature on enzyme activity was determined at each condition set by the Doehlert's design. In this case, temperature varied between 20 and 60°C and pH between 6.0 and 12.0 using the same mixture of buffers described above.

### 2.8. Effect of Inhibitors, Metal Ions, and Organic Solvents on Enzyme Activity

In order to investigate the effect of different inhibitors of proteases, metal ions, and organic solvents on enzyme activity, the crude enzyme was preincubated for 1 h at room temperature with different reagents. Residual enzyme activity was determined and expressed as percentage relative to a reaction control (no addition). The different reagents tested were phenylmethanesulphonyl fluoride (PMSF, 2 mM), iodoacetate (10 mM), ethylenediaminetetraacetate (EDTA, 5 mM), 1,10-Phenantroline (1 mM), and Pepstatin A (100 *μ*g/mL), Ca^2+^, Mg^2+^, Zn^2+^, and Hg^2+^ (1 mM each). The solvents tested were DMSO, ethanol, methanol, and isopropanol (1% v/v each).

### 2.9. Effect of Surfactants and Bleaching Agents on Enzyme Stability

The suitability of the crude protease of *P. lilacinus* as a detergent additive was determined by testing its stability in presence of some surfactants such as SDS (sodium dodecyl sulphate), Triton X-100, and Tween 20, and bleaching agents such as hydrogen peroxide (H_2_O_2_) and sodium perborate. The crude protease was incubated with different concentrations of these additives for 1 h at room temperature (22°C), and then the residual proteolytic activity was measured under standard conditions against a control without any additives, which was taken as 100%.

### 2.10. Detergent Compatibility

The compatibility of the protease activity in crude extract with commercial solid and liquid laundry detergents (locally available) was also studied. The solid detergents tested were Ariel (Procter & Gamble), Drive, Skip, and Ala matic (Unilever), and the liquid ones were Ace and Ariel (Procter & Gamble); also a prewashed liquid named Mr Musculo (SC Johnson & son) was tested.

Solid detergents were diluted in tap water to give a final concentration of 7 mg/mL, and liquid detergents and prewashed were diluted 100-fold to simulate washing conditions [18]. The endogenous enzymes contained in laundry detergents were inactivated by heating the diluted detergents for 1 h at 65°C prior the addition of an aliquot of crude protease. The corresponding reactions mixture were incubated in each detergents mentioned above for 1 h at different temperatures (30–50°C), and the remaining activities were determined under standard conditions. The enzyme activity of a control, incubated under the similar conditions without detergent, was taken as 100%.

### 2.11. Evaluation of Washing Performance

Clean cotton cloth pieces (2.5 cm × 2.5 cm) were soiled with blood: 100 *μ*L of blood without pretreatment was applied to cloth piece and then dried. The stained cloth pieces were subjected to wash treatments with commercial solid detergent (Skip, a solid detergent available in Argentineans' market) diluted in tap water at 7 mg/mL, supplemented with and without crude enzyme. When the wash treatment was with the supplementation of the crude enzyme, endogenous enzymes contained in laundry detergents were inactivated by heating the diluted detergents for 1 h at 65°C prior to the addition of an aliquot of crude protease. 

Two stained cloth pieces were taken in separate flasks, with 50 mL as final volume, as indicated above: flask with tape water, only, flask with tap water and commercial detergent at final concentration of 7 mg/mL, and flask with tap water, commercial detergent, and crude enzyme of *P. lilacinus* (62.5 U_C_/50 mL).

Each flask was incubated at two temperatures: 30 and 40°C for 30 and 60 min under agitation (200 rpm). After incubation, cloth pieces were taken out, rinsed with water, and dried. Visual examination of various pieces showed the effect of the crude enzyme in the removal of stains [19]. 

### 2.12. Statistical Analysis

All analyses were performed at least in triplicate, and data were expressed as means ± standard deviations.

## 3. Results and Discussion

### 3.1. Identification of *P. lilacinus* as Keratinolytic Fungus

**Figure 1:**
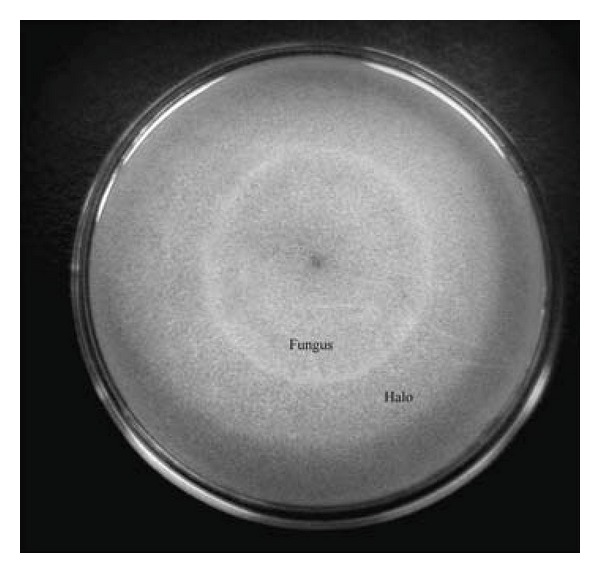
Qualitative test in feather meal agar plates for keratin degrading enzyme activity. A degradation halo surrounding the colony of *P. lilacinus *is observed.

A series of fungal strains of the Spegazzini Institute Fungal Culture Collection (La Plata National University, Argentina) were preliminary screened for their keratinolytic potential using feather meal agar. In our case, *P. lilacinus* (Thom) Samson LPS 876 was selected because, after 15 days of incubation at 28°C, a hydrolysis halo was observed indicating the keratinolytic capability of this strain (Figure 1). The use of this technique in a preliminary screening with a similar purpose was reported by Wawrzkiewicz et al. [20]. Where, among the 16 strains of dermatophytes tested, only *Trichophyton verrucosum* showed tiny fungal colonies surrounded by a wide clear zone of solubilized keratin.

### 3.2. Grow Profile of *P. lilacinus* on Hair Waste Medium

**Figure 2:**
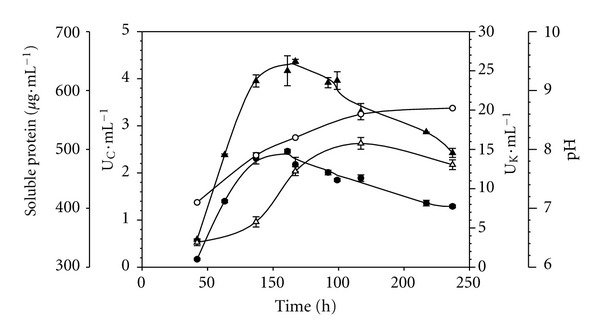
Time course of *P. lilacinus *culture using a minimal mineral medium containing 10 g L^−1^ of hair waste (pH: 7). (●) proteolytic activity; (▲) keratinolytic activity; (*▵*) soluble protein; (◯) pH.

*P. lilacinus* LPS 876 grew well in a minimal mineral medium containing salts and hair waste as sole carbon, nitrogen, and energy source. As can be seen in Figure 2, extracellular enzyme activities (protease and keratinase) were associated with both an increment in soluble protein concentration as well as with a continuous increase in pH values in culture broth. These facts were reported by several authors for other microorganisms with high keratinolytic activity growing on this kind of substrates. The increment in pH value has been pointed out as an important indicator of the keratinolytic potential in microorganisms because of high level of deamination, with the concomitant ammonium accumulation in culture medium [21]. Moreover, Korniłłowicz-Kowlaska and Bohacz [22] concluded that substrate mass loss, a release of peptides and ammonia, sulfate excretion, and substrate alkalinization should be recognized as homogenized (due to mutual correlation) assessment parameters of the keratinolytic activity of fungi.

Enzyme activities were evaluated during the culture time course. Maximum protease and keratinase activities concurred at around 111–117 h of cultivation, (Figure 2). The ratio observed between both enzyme activities (K : C ratio) at different culture times was quite constant (11.28 ± 1.06). Therefore, the presence of a single enzyme responsible of both activities was tentatively postulated. Using the same enzyme assay conditions, it is assumed that a protease with K : C ratio higher than 5 is a true keratinase [23]. Generally, reported keratinases have a K : C ratio ranged from 5 to 20 [14, 15, 24].

The hydrolysis of azokeratin and azocasein by the proteases produced by *P. lilacinus* growing on hair waste was compared with commercial enzymes. The K : C was chosen as criterion for enzyme specificity for keratinous substrates (Table 1). As can be seen, our crude enzyme preparation was superior for hydrolysis of keratin substrate compared with other commercially available proteases. Similar results were reported by Cheng et al. [25] for the keratinase of *Bacillus licheniformis *and by Gradišar et al. [14] for the keratinases of* P. marquandii* and *Doratomyces microsporus*.

### 3.3. Effect of pH and Temperature on Enzyme Stability

In general, all detergent compatible enzymes are alkaline thermostable in nature with a high pH optima because the pH of laundry detergents is generally in the range of 9–12 and varying thermostability at laundry temperatures, (50−60°C) [26–28]. For the study of the effect of pH and temperature a RSM was used. The pH and temperature values used in Doehlert's design for enzyme stability determination are shown in Table 2. The central point was replicated three times in order to determine the experimental error. Data presented in Table 2 were converted into second-order polynomial equation.

Statistical analysis of the results revealed that, in the range studied, the two variables, as well as their interactions, have a significant effect on protease (keratinase) stability.

**Figure 3:**
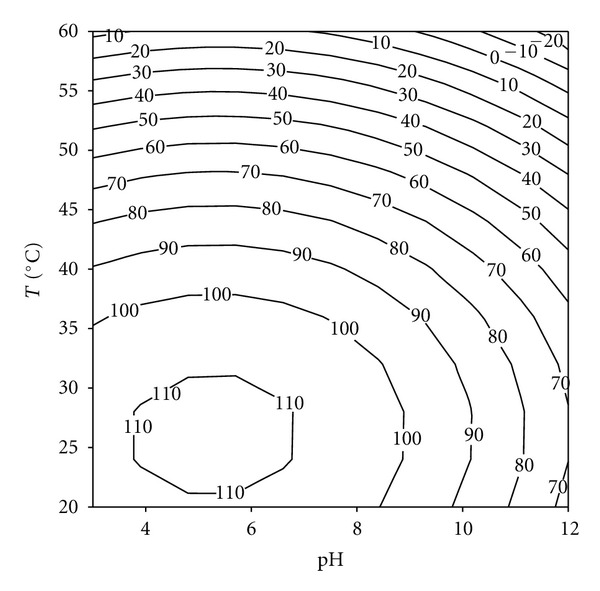
Response surface plot for the effect of pH and temperature on enzyme stability.

The following regression equation was obtained to calculate the percentage of residual enzyme activity (% R.A.) after 2 h of incubation:

(2) (%) R.A.:91.33−18.12∗pH−55.43∗T−18.83∗pH2−46.15∗T2+0.72∗pH∗T,

where pH and *T* are given as codified data. The *r* ^2^ value was equal to 0.91, indicating that only 9% of the total variation was not explained by the model. The contour graph of the proteolytic activity observed as a response to the interaction of pH versus temperature is shown in Figure 3. These results indicate that the enzyme is stable in a wide range of pH and temperatures, preserving more than 60% of its activity after 2 h of incubation at pH 11 and 45°C. A serine proteinase purified from *P. lilacinus *(Thom) Samson VKM F-3891 displayed 40% of residual activity after 3 h incubation at 60°C [29]. However, in other cases, such as the serine proteinase from *Aspergillus chrysogenum*, enzyme stability in the alkaline range is substantially reduced.

**Figure 4:**
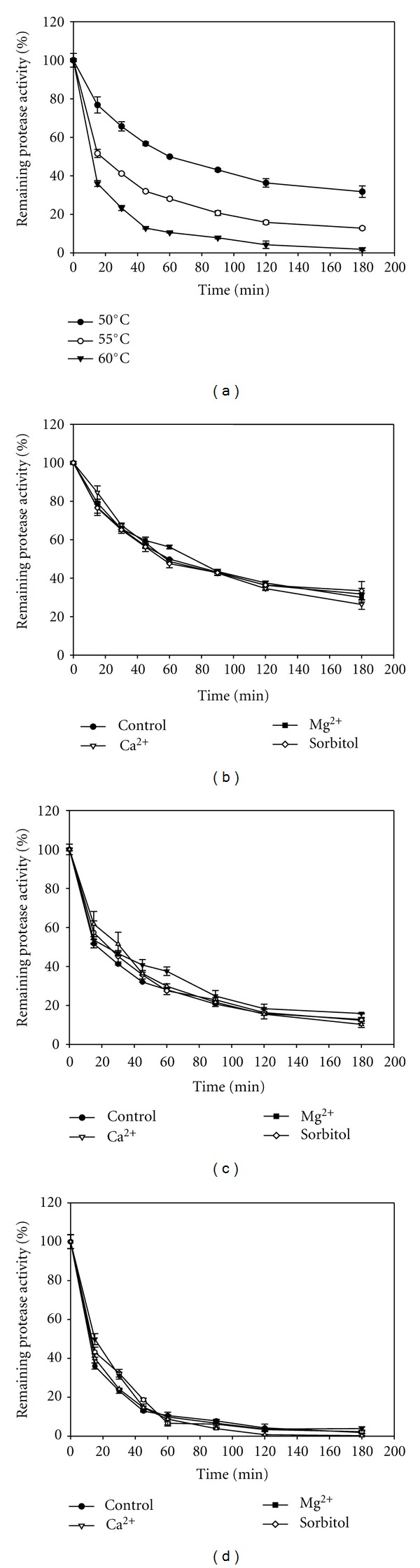
(a) Effect of temperature on protease stability (●) 50°C; (◯) 55°C; (*▾*) 60°C. (b) Effect of stabilizers on heat inactivation at 50°C. (c) Effect of stabilizers on heat inactivation at 55°C. (d) Effect of stabilizers on heat inactivation at 60°C. For (b), (c), and (d) the original activity before preincubation was taken as 100%. Values are means of three independent determinations.

Studies on thermostability of the enzyme at 50, 55, and 60°C revealed that heat inactivation displays a typical first order kinetic (Figure 4(a)). The enzyme exhibited half lives of 62, 29, and 10 min at 50, 55, and 60°C, respectively. Addition of metal ions such as CaCl_2_ and MgCl_2_ (5 mM) individually, improved the thermostability of the enzyme (Figures 4(b) to 4(d)). It can be seen in Figure 4(b), the apparent half-life of the enzyme increased by 1.3-fold, 1.2-fold, and 1.1-fold by the addition of Mg^2+^, Ca^2+^, and sorbitol, respectively. Similar results were observed at 55 and 60°C where sorbitol increased half-life by 1.2-fold. In the case of Mg^2+^, this metal increased the apparent half-life by 1.1-fold and 1.3 fold at 55 and 60°C, respectively. In general, all chemicals tested here produced a slight increase in the thermal stability of the enzyme. 

It had been reported that the addition of Ca^2+^ or polyhydric alcohols, such as glycerol and polyethylene glycol caused an increase in thermal stability of alkaline proteases. The addition of sorbitol improved the thermal stability for an alkaline protease from *B. cereus* BG1, which increased its thermal stability by approximately 2-fold at 60°C [30]. Kelkar and Deshpande [31] studied the influence of various polyols on the thermostability of pullulan-hydrolysing activity from *Sclerotium rolfsii*. The half-life of the enzyme activity at 60°C was about 30 min, and in presence of xylitol and sorbitol (3 M) they reported a significant enhancement in the thermostability of the enzyme with retention of 100% activity after incubation for 7 h at 60°C. Ghorbel et al. [30] reported an alkaline protease that, in the presence of 10mM Ca^2+^, retained 100, 93, and 26% of its initial activity after heating for 15min at 55, 60, and 70°C, respectively. However, the enzyme was completely inactivated when incubated at 55°C for 15 min in the absence of calcium. On the contrary, the enzyme reported here in presence of 5 mM Ca^2+^ retained about 57 and 43% of its initial activity after heating 15 min at 55 and 60°C, respectively, but the enzyme in absence of calcium retained more than 25% of its initial activity after 60 min of incubation at 55°C.

Several reports showed that the addition of various additives such as polyols and PEG could enhance enzymes' thermal stability [2, 18]. The increase in the thermal stability by adding these such as additives was probably due to the reinforcement of the hydrophobic interactions among nonpolar amino acids inside the enzyme molecules and thus increased their resistance to inactivation, since it had been reported that polyols modify the structure of water and/or strengthen hydrophobic interactions among nonpolar amino acids inside the protein molecules [32].

The effect of Ca^2+^ on the improvement of thermal stability against heat inactivation may be explained by the strengthening of interactions inside proteases molecules and by binding of this metal to the autolysis site. The activity of *B. mojavensis* protease was enhanced not only by Ca^2+^ but by Fe^2+^ and Mn^2+^. It is believed that metal ions protect the enzyme against thermal denaturation and play a vital role in maintaining the active conformation of the enzyme at higher temperatures [33].

### 3.4. Effect of pH and Temperature on Enzyme Activity

The pH and temperature values used in Doehlert's design for determination of the effect of pH and temperature on enzyme activity are given in Table 3. 

**Figure 5:**
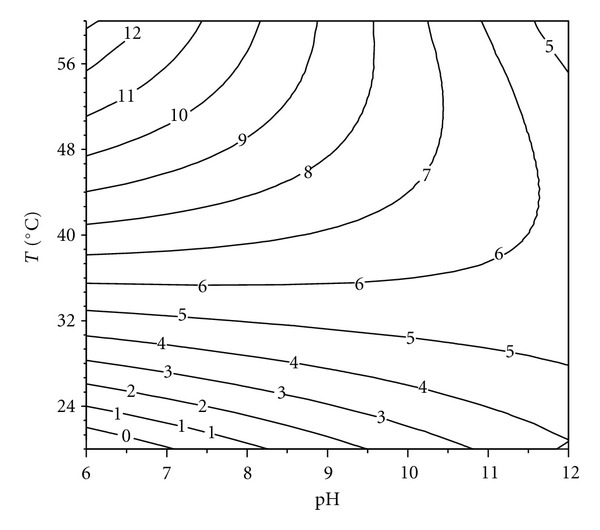
Response surface plot for the effect of pH and temperature on enzyme activity.

The following regression equation was obtained to calculate the enzyme activity (E.A):

(3) EA (U·mL−1):6.48−0.928∗pH+4.12∗T−0.188∗pH2−2.23∗T2−3.835∗pH∗T,

where pH and *T* are given as codified data. The *r* ^2^ value was equal to 0.89, indicating that only 11% of the total variation was not explained by the model. The contour graph of the keratinolytic activity observed as a response to the interaction of pH versus temperature is shown in Figure 5. These results indicate an optimal pH and temperature of 6.0 and 60°C, respectively. Kotlova et al. [29] reported a thiol-dependent serine proteinase from *P. lilacinus* (Thom) Samson VKM F-3891 with an optimal pH into the alkaline range. Such pH dependence is also reported by Bonants et al. [34] for another proteinase isolated from a different strain of the fungus*, P. lilacinus *(Thom) Samson (CBS 143.75). These proteinases seem to belong to the subgroup of subtilisin-like enzymes with an optimum pH in the alkaline range (pH 10–12).

 In our case, the protease present in the enzyme extract belongs to the group of enzymes, with an optimum pH in a neutral range (pH 6–8) at an assay temperature of 60°C. Although optimal temperature for keratin degradation is 60°C, the stability of the enzyme under this condition is not so high. Because of that, a temperature of 55°C, where the enzyme retains more than 90% of the optimal activity, is proposed for practical applications.

### 3.5. Effect of Protease Inhibitors, Metal Ions, and Organic Solvents on Enzyme Activity

The effect of various chemical reagents and metal ions on enzyme activity with azocasein as substrate is shown in Table 4. The enzyme activity was strongly inhibited by PMSF (93% of inhibition), a well-known inhibitor of serine proteinases, in particular subtilisins serine proteinases [35]. It was slightly inhibited by Pepstatin A but neither by iodoacetate, EDTA, nor by 1,10-Phenantroline. These facts suggest that in our case, the enzyme produced by *P. lilacinus* corresponds to a serine protease. Actually, most keratinases described until now are classified into this category [36]. The stability of the enzyme in presence of EDTA is advantageous for its use as a detergent additive. An enzyme, which is to be used as a detergent additive, should not have requirement for a metal cofactor. This is because detergent formulations contain high amounts of chelating agents, which specifically bind to and chelate metal ions making them unavailable in the detergent solution. The chelating agents remove the divalent cations responsible for water hardness and also assist in stain removal.

Among the metal ions tested, Hg^2+^ strongly inhibited proteolytic activity (94% of inhibition), whereas Ca^2+^ and Mg^2+^ caused slight activation. Hg^2+^ is recognized as an oxidant agent of thiol-groups, and the enzyme inhibition by this ion suggests the presence of an important-SH group (such as free cysteine) at/or near the active site [37]. In addition, the strong inactivation by Hg^2+^ is typical for proteinases belonging to the thermitase and proteinase K subgroups [38]. This feature makes our *P. lilacinus* keratinase substantially different to the true bacillary subtilisins, as well as to the serine proteinase from *P. lilacinus* isolated by Bonants et al. [34], which is not inactivated by Hg^2+^. Based on the presence of functionally important sulfhydryl groups, our keratinase resembles proteinase K and bacillary thiol-dependent subtilisins much more than other fungal serine proteases.

Divalent metal ions such as Ca^2+^ and Mg^2+^ slightly activated the enzyme. This fact could be explained because of that they could act as salt or ions bridges stabilizing the enzyme under its active conformation, and thus they might protect the enzyme against thermal denaturation [39, 40].

Crude enzyme preparation showed to be highly stable in presence of different organic solvents such as DMSO, ethanol, methanol, and isopropanol (Table 4), a positive fact considering the potential practical application.

### 3.6. Effect of Surfactants and Bleaching Agents on Enzyme Stability

The above-mentioned characteristics of our *P. lilacinus* protease suggested its potential use in different applications like laundry detergent formulation. In order to be effective during washing, a good detergent protease must be compatible and stable with all commonly used detergent compounds such as surfactants, bleaching agents, and other additives, which might be present in detergent formulation [1]. In our case, a crude protease extract was incubated 60 min at room temperature in presence of several additives, and then the residual protease activity was assayed under standard conditions. 

As can be seen in Table 4, crude protease was highly stable in presence of nonionic surfactants. It retained near 100% of its initial activity in presence of 0.5% Triton X-100 and 0.5% Tween 20. In presence of 0.5% of SDS, a strong anionic surfactant, it exhibited moderated stability (75%) after 1 h of incubation. SDS is known to be a strong denaturant of proteins including alkaline proteases. It could unfold most proteins through the interaction between the charged head group of SDS and the positively charged amino acid side chains of proteins and between the alkyl chain of SDS and the nonpolar parts on the surface as well as in the interior of proteins [41]. The retention of protease activity by our enzyme preparation in presence of SDS was higher than that of a protease from *Aspergillus clavatus* ES1, which retained only 33% of its activity under the same stability assay conditions [42].

On the other hand, our enzyme preparation showed excellent stability toward bleaching agents such as H_2_O_2_ and sodium perborate (Table 4). It showed an stability similar to proteases from *B. licheniformis* NH1, which retained 85% and 80% of its activity after incubation with 0.5% H_2_O_2_ [19] and resulted in being more stable than an alkaline protease from *B. licheniformis* RP1 [43] which is less stable against bleaching agents; it's just retained 68% and 48% of its activity after 1 h incubation at 40°C in presence of 2% H_2_O_2_ and 0.2% sodium perborate, respectively. Bleaching agents inactivate proteins oxidatively, being Met the primary site for oxidative inactivation. All subtilisins contain a Met residue next to the catalytic Ser residue, so that many of them tend to undergo oxidative inactivation in presence of a bleaching agent such as hydrogen peroxide. Thus, many of available alkaline proteases exhibited low activity and stability toward oxidants, which are common ingredients in modern bleach-based detergents. To overcome these shortcomings, several attempts have been made to enhance enzyme stability by protein engineering [44]. That is why it is important to obtain enzymes with high stability against surfactants and oxidants for practical applications. Detergent applications for keratinases have been also suggested [36]. These include removal of keratinous dirt that are often encountered in the laundry, such as collar of shirts and used as additives for cleaning up drains clogged with keratinous waste.

### 3.7. Detergent Compatibility

All the commercial detergents contain hydrolytic enzymes, and these enzymes-based detergents known as “green chemicals” find a wide range of applications in laundry, dishwashing, textile, and other related industries [45].

**Figure 6:**
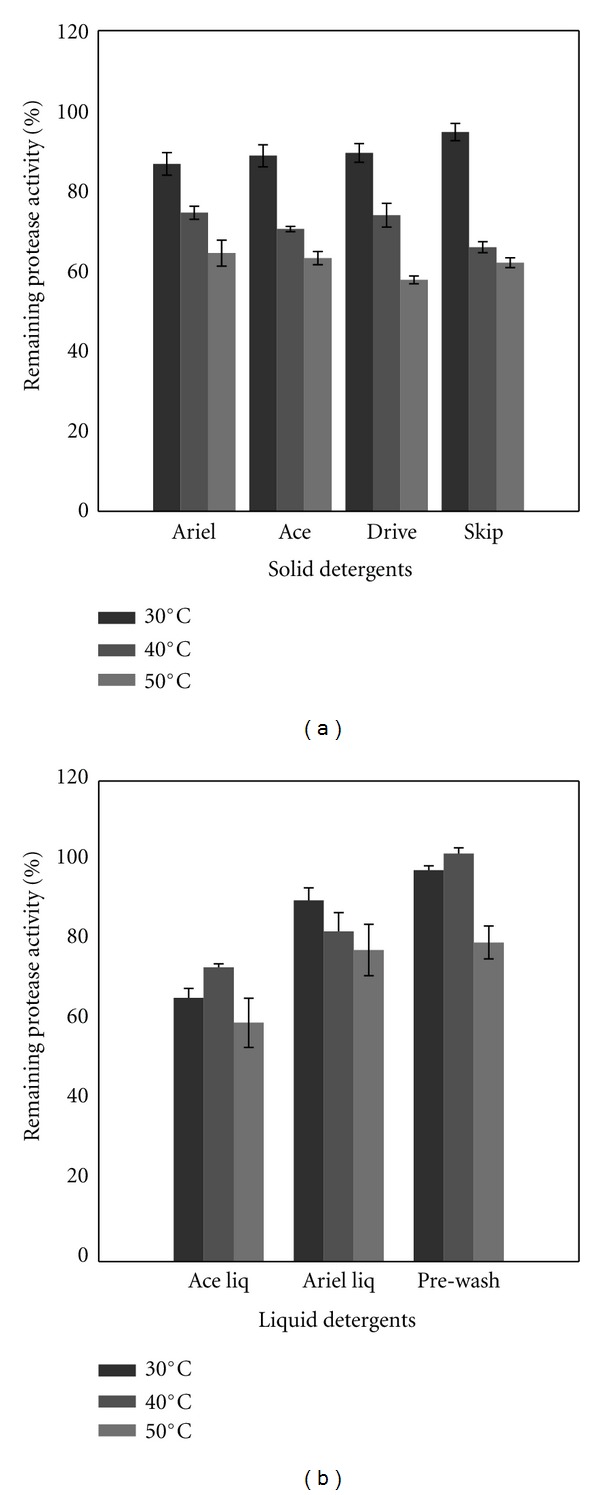
Stability of the crude enzyme in the presence of various commercial solid (a) and liquid detergents (b). CE was incubated in each detergent mentioned for 1 h at different temperatures (30–50°C), and the remaining activities were determined under standard conditions. The enzyme activity of a control, incubated under similar conditions without detergent, was taken as 100%.

In order to check the compatibility with liquid and solid detergents, the crude enzyme was preincubated in presence of various commercial laundry detergents for 1 h at 30, 40, and 50°C. Solid detergents were diluted in tap water to a final concentration of 7 mg/mL, and the liquid ones were diluted 100-fold to simulate washing conditions. As can be seen in Figure 6(a) the crude enzyme was very stable towards all solid detergents tested, even at 50°C after 1 h of incubation, it retained more than 60% of its activity in presence of Ariel, Ace, and Skip. In presence of Drive, it retained about 57% of its activity being more stable than an alkaline protease from *Vibrio fluvialis* strain VM10 reported by Venugopal and Saramma [46], which retained just 42% of its activity in presence of Ariel as well as an alkaline serine protease from *Bacillus sp*. SSR1 reported by Singh et al. [47] which retain lees than 40% of its activity after 1 h of incubation in presence of Ariel at 40°C. Interestingly, it was more stable than the commercial protease named Maxacal, which retained less than 60% after 1 h of incubation in presence of Ariel at 40°C [47]. Similarly, proteases from *B. mojavensis* A21 [48] and from *B. licheniformis* RP1 [43] are shown to retain more than 40 and 80% of their activity in presence of Dixan after 1 h of incubation at 50°C, respectively.

In presence of liquid detergents, the crude enzyme retained more than 75% and 50% of its initial activity in presence of Ariel and Ace, respectively, after 1 h of incubation at 50°C (Figure 6(b)). 

From the results presented here about the compatibility and stability whit commercial detergents at different temperatures, it can be concluded that our protease will be more effective at temperature from 30 to 40°C for long washing cycles (60 min) and at 50°C for short washing cycles (10–30 min). But with Ariel liq. long washing cycles could be done at 55°C too, because it retained about 78% of its original stability.

### 3.8. Wash Performance Analysis

**Figure 7:**
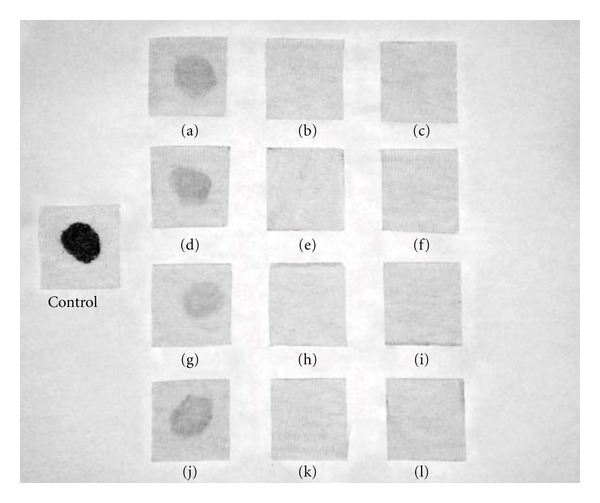
Washing performance analysis of the *P. lilacinus *enzyme preparation in the presence of the commercial detergent Skip. Analysis were done at 30°C ((a)–(f)) and 40°C ((g)–(l)), for 30 m ((a)–(c) and (g)–(i)) and 60 m ((d)–(f) and (j)–(l)). Cloth washed with tap water ((a), (d), (g), and (j)); ((b), (e), (h), and (k)) cloth washed with Skip; ((c), (f), (i), and (l)) cloth washed with Skip added with crude enzyme of the *P. lilacinus *protease.

The wash performances of the protease present in the crude extract were assessed by its ability to remove blood stain from white cotton cloth (Figure 7). Enzyme in combination of the commercial detergent Skip was tried. The visual comparison of the washed cloth revealed that washing with distilled water at temperatures of 30 and 40°C removed some amount of blood stain from the cotton cloth (Figures 7(a), 7(d), 7(g), and 7(j)). As can be seen, the replacement of the enzymes present in the commercial detergent by the crude enzyme gave a complete blood stain elimination at both temperatures and times, such as the endogenous enzymes from the commercial detergent has done (Figures 7(c), 7(f), 7(i), 7(l)). These results show the efficiency of *P. lilacinus *protease in proteinaceous stain removal efficiency.

Abidi et al. [49] showed also the significant improvement of the supplementation of proteolytic preparation of *Botrytis cinerea*, in a laundry detergent (Henkel-Alki), in the elimination of blood, egg yolk, and chocolate stains on fabric. Jellouli et al. [50] and Savitha et al. [51] reported similar results in their wash performance tests. Therefore, *P. lilacinus *crude extracts containing protease activity could be considered as a potential candidate for use as cleaning additive in detergents to facilitate the release of proteinaceous stains.

## 4. Conclusions

A locally isolated *P. lilacinus* strain produces an extracellular protease with keratinase activity when grown on hair waste, the main solid-wastes produced in tanneries, as substrate in submerged cultures. 

The protease was characterized, and it exhibited remarkable stability toward surfactants, bleaching agents, and detergent additives like EDTA and sodium perborate. This property of the enzyme is very essential for its application as detergent additive. Our study shows that the extracellular proteolytic enzyme produced by this strain could have an industrial application in detergent industries. Moreover, the enzyme was compatible with most of the laundry detergents tested and showed a good washing performance.

## Figures and Tables

**Table 1:** Specific proteolytic activity (U_C_/mg), keratinolytic activity (U_K_/mg), and K : C ratio of crude extract and commercial proteases.

	Proteolytic activity (U_C_/mg)	Keratinolytic activity (U_K_/mg)	K : C ratio
Enzyme preparation	15.67	159.3	10.17
Proteinas K	33.9	218.7	6.45
Alcamax	4.6	27.4	5.96
Papain	7.6	3.9	0.51

**Table 2:** Actual values (experimental data) and residual activity attained in Doehlert's design for pH and temperature stability.

	pH	Temperature (°C)	Residual activity (%)
	Experimental data		
A	7.5	40	93.4
A	7.5	40	89.1
A	7.5	40	92.2
B	12	40	45.1
C	9.75	60	3.25
D	5.25	60	4.5
E	3	40	104.4
F	5.25	20	106.4
G	9.75	20	104.4

**Table 3:** Actual values (experimental data) and enzyme activity (U_C_·mL^−1^) attained in Doehlert's design for the study of the effect of pH and Temperature on enzyme activity.

	pH	Temperature (°C)	U_C_·mL^−1^
	Experimental data		
A	9	40	6.5
A	9	40	6.4
A	9	40	6.5
B	12	40	6.6
C	10.5	60	5
D	7.5	60	11.6
E	6	40	6
F	7.5	20	1.2
G	10.5	20	1.2

**Table 4:** Effect of protease inhibitors, metal ions, detergents, and solvents on protease activity (data are given as Residual activity (%) ± SD).

Chemical none	Concentration	Residual activity (%) 100
Inhibitor		
PMSF	2 mM	7.0 ± 0.0
Iodoacetate	10 mM	95.1 ± 4.7
EDTA	5 mM	99.6 ± 6.3
1,10-Phenantroline	1 mM	100 ± 0.2
Pepstatin A	100 *μ*g/mL	87.5 ± 5.5
Metal ion		
Mg^2+^	1 mM	105.0 ± 1.2
Zn^2+^	1 mM	92.8 ± 1.4
Ca^2+^	1 mM	102.9 ± 0.5
Hg^2+^	1 mM	6.0 ± 0.6
Detergents		
Triton X-100	0.5% (v/v)	97.7 ± 2.7
Tween 20	0.5% (v/v)	98.5 ± 2.0
SDS	0.5% (v/v)	75.9 ± 3.4
Bleaching agent		
	1% (w/v)	140 ± 2.3
H_2_O_2_	2%	137 ± 0.3
	3%	122.7 ± 4.0
		
	0.2% (w/v)	99.7 ± 2.4
Sodium perborate	0.5%	97.6 ± 0.9
	1.0%	90.8 ± 2.0
Solvent		
DMSO	1% (v/v)	106.0 ± 1.6
Ethanol	1% (v/v)	105.4 ± 4.0
Methanol	1% (v/v)	117.0 ± 2.9
Isopropanol	1% (v/v)	98.9 ± 1.7
